# A new approach to determine the capture conditions of bark beetles in pheromone-baited traps

**DOI:** 10.1080/13102818.2014.974015

**Published:** 2014-11-12

**Authors:** Gonca Ece Ozcan, Osman Cicek, Korhan Enez, Mustafa Yildiz

**Affiliations:** ^a^ Department of Forestry Engineering, Faculty of Forestry, Kastamonu University, 37150Kastamonu, Turkey; ^b^ Department of Electric and Energy, Arac Rafet Vergili Vocational School of Higher Education, Kastamonu University, 37800Kastamonu, Turkey; ^c^ Tosya Technical Occupation and Vocational High School, 37300Kastamonu, Turkey

**Keywords:** pheromone-baited traps, bark beetles, electronic control unit, solar energy, photovoltaic systems

## Abstract

Forests form an organic unity with a great number of organic and inorganic components and tend to maintain the sustainability of their existing balance. However, some factors which adversely affect the balance of nature may interrupt this sustainability. The epidemic which is formed by bark beetles in their spreading region, due to various factors, changes the stability so much that interference is required. One of the most common methods used to monitor these beetles is pheromone-baited traps. The recognition of parameters, such as date (day/month/year), temperature and humidity, when bark beetles are captured in pheromone-baited traps, especially those used for monitoring will help to increase the trap efficiency on land and to develop an effective strategy for combating pests. In this study, an electronic control unit was added to pheromone-baited traps in order to obtain all of the above mentioned parameters. This unit operates with microcontrollers and data related to the parameters is saved in a storage unit. This is triggered by the beetle at the moment it is captured in the trap. A photovoltaic system was used to meet the energy needed for the system functioning and to complete the counting process in due time.

## Introduction

In parallel with global developments, factors such as beetle damages, fires, natural disasters, global warming, wind throws and snow throws, unsuccessful forestry practices, infrastructure investments, allocation of forest areas for tourism and agricultural activities, illegal grazing and illegal logging adversely affect our forest existence and the quality of forests in Turkey. As a part of the forests great ecosystem, bark beetles may bring significant damage by forming epidemic in their spreading region.

Some beetles and/or pathogens cause more losses to forests than other factors, including fire damage.[1] Bark beetles of *Dendroctonus*, *Ips* and *Scolytus* species are the most harmful beetles in the forests of the northern hemisphere.[2] Bark beetle populations may be exposed to effective rises and falls [3] and pose a threat for forests when their populations exceed a certain threshold.[4] They infest few of the trees at low population level, while they may suddenly damage many trees through an epidemic.[3,5] *Dendroctonus micans* (Kugelann), *Ips sexdentatus* (Boerner), *Ips typographus* (Linnaeus), *Cryphalus picea* (Ratz.), *Pityokteines curvidens* (Germ.), *Ips acuminatus* (Gyll.), *Tomicus piniperda* (L.), *Tomicus minör* (Htg.) and *Orthotomicus erosus* (Woll.) caused significant losses in forests in Turkey and the damage of those beetles result in the drying of tens of thousands of square metres of trees every year.[6–16] It is a necessity to develop a long-term applicable control programme and strategies in order to prevent the losses and destructions caused by these beetle species.[17] Taking annual control precautions reduces the severity of the damage caused by bark beetles.[18] The main objective of a control programme against bark beetle outbreaks is to reduce the damage caused by this outbreak on stands significantly.[19] The use of pheromone is one of the most promising technique against pests [19,20] and aggregation pheromones of many infesting bark beetle species are found and used commercially.[21] Pheromone traps are used to monitor bark beetle populations [4,21–24] and they can help to support suppression of especially low-density populations and to obtain data about population fluctuations.[25] Furthermore, they can be useful to assess damage risk of bark beetles.[4] Expenses in combating activities against bark beetles bring a significant economic burden,[26] and it is accepted that the opportunity to use pheromone traps in combating pests will be economical.[27] However, it is hard to suppress local populations of bark beetles by means of pheromone traps.[28] Monitoring based on pheromone traps may give information about number and flight periods of beetle captures, which might depend on the density of beetle population year over year.[29] The most important property expected from a pheromone trap is to spread out pheromone continuously with the closest rate to the most effective amount during its lifetime,[30] to capture as many flying beetles as possible and to reduce population density and outbreak risk.[19]

Flying behaviour of beetles is a very complex phenomenon [31] and pheromone traps are applied in the spring and summer months, when beetles fly to search host trees.[29] Capture capacities of traps are affected by a number of factors, such as being hung at appropriate places on the trees to enable beetles to reproduce, being close to the infested trees, stand density, wind direction,[32] pest biology,[33] etc.; in addition to trap model, pheromone quality and amount, distribution area, capture period and other factors affect the number of beetles to be captured.[34] The number of bark beetles captured in pheromone traps may differ by spatial differences related to the characteristics of the habitat and ecologic factors.[25] Temperature conditions are very important for the development of bark beetles.[34] On condition that climate changes result in warmer vegetation periods, bark beetles will play a more important role on forest dynamics.[35] Beetles can find pheromone source only under average air and climate conditions,[36] while daily temperatures affect capture rate of beetles in traps, and fluctuations occur not only in temperature values but also in the number of beetles captured in traps during flight period.[34]

Pheromone-baited traps which are used in forestry practices today serve to limited capture of pest species and monitoring of their population. Beetles are captured in traps during their flight periods in regard to their population and climate conditions in the spreading region. However, effective pheromone conditions at the moment when the pest is captured in the trap are not known completely. Traps can be controlled at intervals of 7–10 days and collective results of captures within this period are obtained. It is important to detect flying dates (day, month, hour) and density of flying conditions in details.

In addition, to count, the number of captured beetles in a long term with a labour intensive method increases workforce of applier in existing practices and adversely affects the reliability of results. The reflection of developing technology on forestry practices is getting more and more important today. In existing practices, there is no mechanism to determine capture time of the beetles, and the temperature and humidity parameters at the capture moment. To make these detections will contribute to monitor population density with regards to these parameters in the region where the species spread and to develop an effective strategy to combat against the pest. To this end, electronic control unit (ECU) was established and a microcontroller is used to save parameters at the moment when each beetle enters the storage unit of a pheromone-baited trap.

## Materials and methods

In our technology, renewable energy sources (RES) can be used for electricity generation, because they are reliable and can meet the energy need. Being one of the RES, photovoltaic (PV) technology has become one of the most promising alternatives for the use in energy technology.[37] PV systems are used in various fields, such as telecommunication, cooling, water pumping (especially in agricultural irrigation), in order to meet energy need in rural areas, etc.[38] PV systems can be divided into four categories by the main practices: off-grid domestic PV systems, off-grid non-domestic PV systems, grid-connected distributed PV systems and grid-connected centralized photovoltaic systems.[39] Off-grid domestic PV systems are preferred to ensure uninterrupted energy needed by ECU under land conditions.

In addition to PV modules, necessary components to design a PV system are battery charge controller, battery, inverter, security switch and fuses, earth return circuit and cabling units.[40] Of PV system equipments, PV module is used to generate electricity from sunlight;[41] battery charge controller is used to transfer the electric energy received from PV module to batteries and system in control;[42] battery is used to store electric energy received from PV module after it passes to battery charge controller and to use stored energy during day and night;[43] security switch and fuses are used to ensure the security of the system.[44] In the designed system, no inventor was used as there was no alternating current (AC) load. The aim is to determine parameters at the capture moment of beetles in the pheromone trap.

To this end, ECU is used to save temperature and humidity variables at the trap capture moment of beetles, which are perceived by the sensor in a micro-SD card. The saved parameters are storage as comma separated value (CSV) file within the micro-SD card by microcontrollers. The designed unit can be applied for all trap types as it is placed at the input of the trap chamber. In this study, three Scandinavian horn pheromone-baited traps®, which are commonly used in Turkey to monitor bark beetles, were chosen. PV system equipments (PV module, battery, charge controller, fuse and cabling) and electronic card equipments (microcontrollers, sensors, micro-SD card and electronic units) were used to form ECU.

### Electronic control unit (ECU)

**Figure 1.**
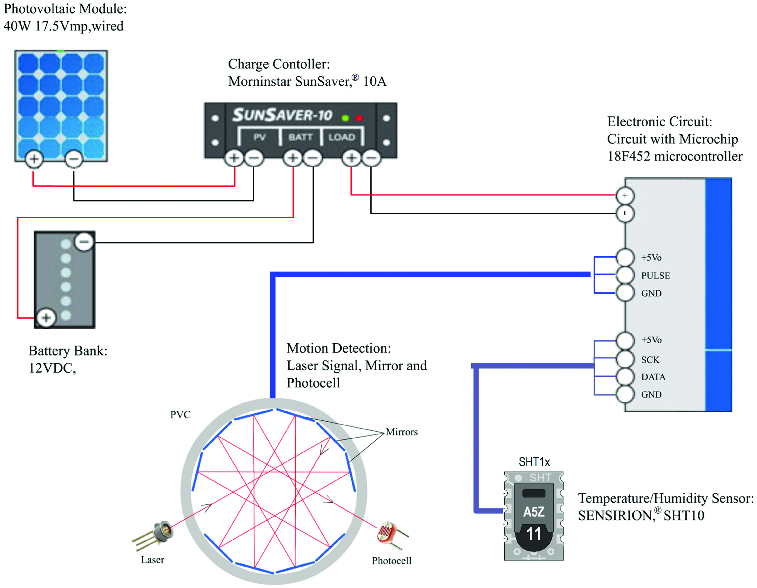
Block diagram of electronic control unit.

ECU is needed to define instant values of parameters to be saved at the capture moment of beetles in the trap. There are various microcontrollers that can be used for this purpose.[45–50] Considering properties and costs in this line, Microchip company's PIC18F452 microcontroller was preferred. This microcontroller controls storage unit with its MSSP (Master Synchronous Serial Port) feature. In addition, it can make keypad, sensor and LCD controls simultaneously through its other input/output ports.[50] The block diagram of ECU which was formed with PV system and electronic system equipments is presented in Figure 1.

**Figure 2.**
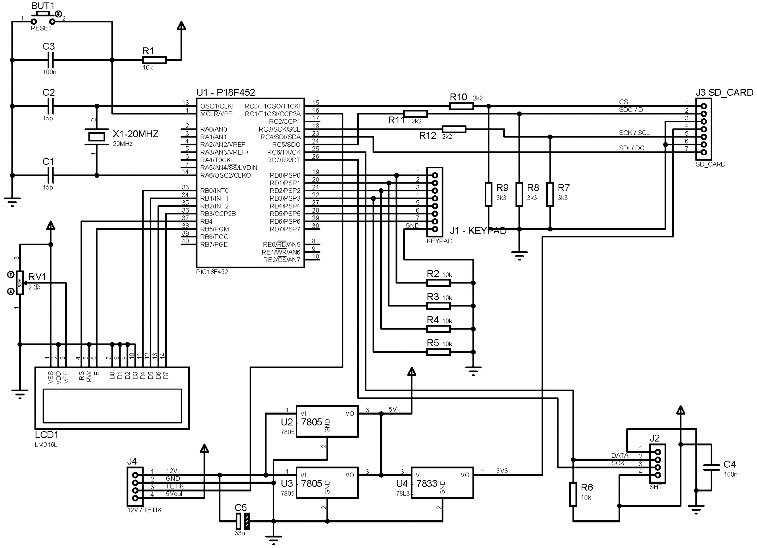
Circuit scheme of electronic card.

The energy stored in accumulators is used to meet the ECU energy need when the sun is not effective due to the off-grid design of the PV system and the PV module. A charge control unit was used to store electric energy generated by the PV module in 12 V accumulators. Regulator circuits were used to meet 3.3 V voltage need of the information storage unit and 5 V voltage need of the electronic card to function. A 2×16 LCD screen was used to obtain information about the situation of applicator and to monitor arrangements that might be needed during the application of traps in the forest. Within normal operation range, this screen helps to display instant date, hour, humidity and temperature information. Installation settings of traps (pheromone number, date, hour) are determined with a keypad. At this point, the keypad also provides a variety of controls through a microcontroller input/output port. Micro-SD memory card was used for storage of parameters to be monitored. Circuit scheme of the electronic card is presented in Figure 2.

### System parameters and sensors

**Figure 3.**
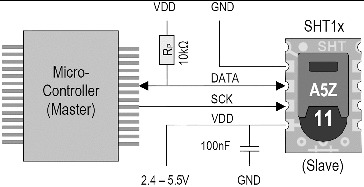
Typical application circuit of SHT1x® sensors.[53]

It is aimed to obtain temperature, humidity, date and hour parameters at the moment of capture in the pheromone trap during flying time and periods of pest species by means of this mechanism. Code blocks which are placed in microcontrollers are used to find date (day/month/year) and hour information. There is a variety of sensors to measure temperature and humidity parameters. There are also some sensor types which can indicate these temperature and humidity information simultaneously.[51–53] SHT10® sensor was used in this study as it indicates these information simultaneously and digitally. This sensor digitally indicates 14-bit temperature and 12-bit humidity information as default values.[53] Typical application circuit of SHT1x® sensors is presented in Figure 3.

### Storage of parameters

**Figure 4.**
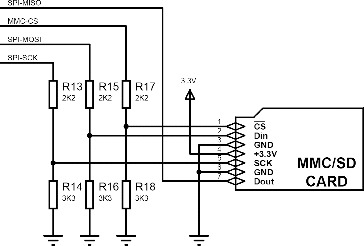
Typical application circuit of micro-SD sensors.[54]

It is important to determine population density of the pest species in the spreading region and parameters at the moment of trap capture. Therefore, it is necessary to obtain the saved parameters at the capture moment of beetles in the trap, without any interruption. Micro-SD card was used in experimental mechanism due to its low energy consumption, being easy to mount and because of the easy communication methods between microcontrollers. Micro-SD card uses MSSP (serial peripheral interface [SPI] mode) method in communication with the microcontroller through three links. According to this method, typical application diagram of micro-SD card is presented in Figure 4.[54]

**Figure 5.**
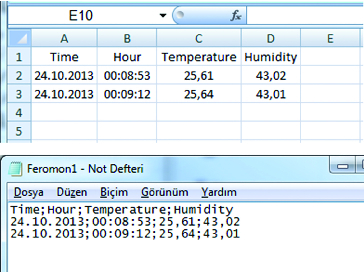
Print screen of CSV.

In order to store data on micro-SD card, file type of memory card should be arranged as a Fat16 (exFat) filing system, which can be supported by the microcontroller. The obtained data should be stored in table form, to be organized and clear (Figure 5). In this study, the data is stored as ‘Comma separated CSV’ file type within storage unit by microcontroller.[55]

### Perceiving beetles captured in pheromone-baited trap

Sensation is the most efficient section which determines the number of pest species captured in the pheromone trap and triggers other units in the system within ECU. There are many sensors which function on different bases, for example, infrared, ultrasonic, PIR (motion) etc.[56–59] It is assumed that error margin of these sensation methods in perceiving captured beetles would be high, as these beetles have millimetric sizes. In this study, it is assumed that laser systems, which are commonly used especially in security fields, would minimize the error margin through perceiving that linear light transmission is interrupted by an object.

**Figure 6.**
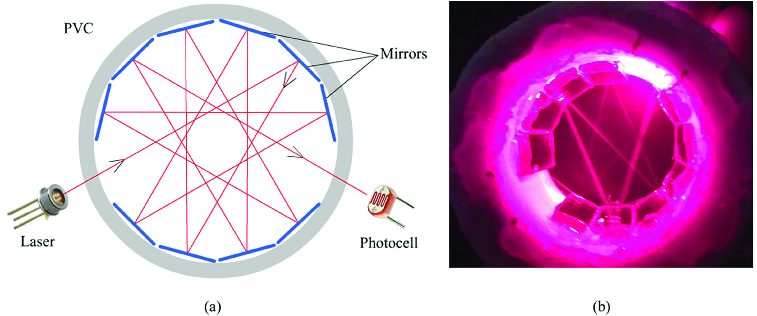
(a) Design of laser scanning application. (b) Real image of laser scanning application.

The presence of linear laser light is continuously monitored with a photocell. With the interruption of this light transmission by a beetle inside the trap, the sensation process is completed (Figure 6(a) and 6(b)). This interruption is the signal for recording the date, time and conditions of the capture. PV system was used to meet energy need for the system functioning and to complete the counting processes in due time.

## Results and discussion

**Figure 7.**
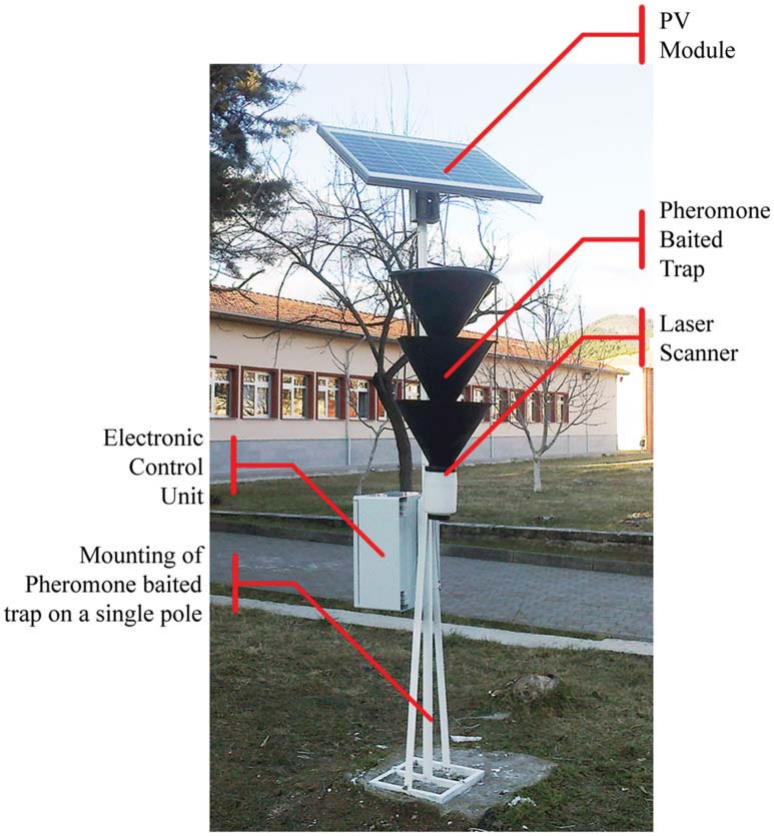
Design of pheromone-baited traps with electronic control unit.

It is important to use pheromone traps in the most efficient way against bark beetles, which cause significant damages in forests in Turkey. To this end, ECU will enable to determine date, temperature and humidity parameters at the capture moment of pests in traps during their flight period and times. Thus, the reliability of counting results will increase and labour force of applicators will decrease significantly. The results to be obtained will contribute to monitor population density with regards to these parameters in regions where the pest species spread and to develop efficient combating strategies against pests in forestry field. Experimental mechanism of the ECU structure, which was designed in line with the mentioned aims, is presented in Figure 7.

**Figure 8.**
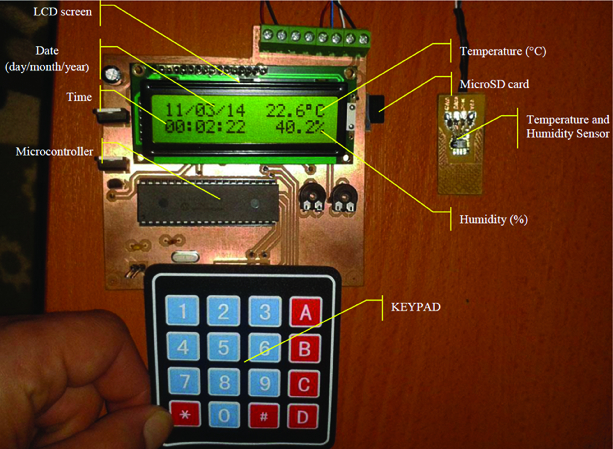
Electronic control card.

In ECU mechanism, an electronic control card was designed to determine parameters at the capture moment of beetles in the trap and to store those parameters on a micro-SD card. The figure of this structure is presented in Figure 8.

Laser was used to detect the moment when the beetle reaches the trap chamber on pheromone trap structure. According to the size of insects, species specialization will be focused on in the next version.

PV system was used to meet the energy need for the functioning of ECU and to complete the counting process in due time. PV system was preferred for energy need as pheromone traps would be placed in sun-soaked places during sunshine duration, such as in-forest spaces within application area.

In following stage, ECU pheromone-baited traps will be applied on the land, and application results based on the measurement and counting will be assessed. This projection is the first study which has been designed to obtain data from pheromone traps, which are being used to integrate technologic developments in forestry activities in Turkey.
